# Structure-function models of temporal, spatial, and spectral characteristics of non-invasive whole brain functional imaging

**DOI:** 10.3389/fnins.2022.959557

**Published:** 2022-08-30

**Authors:** Ashish Raj, Parul Verma, Srikantan Nagarajan

**Affiliations:** Department of Radiology and Biomedical Imaging, University of California, San Francisco, San Francisco, CA, United States

**Keywords:** brain activity, fMRI, MEG, EEG, neural mass model, spectral graph theory, Laplacian, structure-function models

## Abstract

We review recent advances in using mathematical models of the relationship between the brain structure and function that capture features of brain dynamics. We argue the need for models that can jointly capture temporal, spatial, and spectral features of brain functional activity. We present recent work on spectral graph theory based models that can accurately capture spectral as well as spatial patterns across multiple frequencies in MEG reconstructions.

## 1. Introduction

One of the outstanding questions in the field of neuroscience is to understand how dynamic brain functional activity arises despite being constrained by the static anatomical structure (Bassett and Bullmore, 2009; Cao et al., 2014; Fornito et al., 2015; Suarez et al., 2020). To explore this relationship, both structure and functional activity are estimated from non-invasive neuroimaging techniques such as magnetic resonance imaging (MRI), functional MRI (fMRI), and magneto-/electroencephalography (M/EEG). Given that such techniques cannot capture the underlying mechanistic biophysics of the brain activity, computational approaches are widely used to fill this gap. Many of these approaches are based on interpreting the brain structure as a graph with nodes as different brain regions and graph edges as the white matter fibers connecting the brain regions. Subsequently, various graph-based statistical as well as mathematical modeling techniques have focused on capturing the temporal, spectral, and spatial patterns of the brain in different brain states as well as diseases.

A key question in computational neuroscience is the elucidation of the exact relationship between the regional neural signals and their functional connectivity (FC) organization on one hand, and the underlying structural or anatomic connectivity (SC) on the other. This is what is referred to as the *structure-function relationship* in network neuroscience. Although these relationships may be addressed using purely statistical tools e.g., by evaluating the correlation between SC and FC (Hagmann et al., 2008), it is far more elegant to explore these relationships using biophysical mathematical models to serve as a formal interface between the physiology of the brain and its anatomic and connectivity properties (Honey et al., 2010). Moreover, mathematical models can be used for exploring biophysics underlying the whole brain imaging activity, and identify biophysically interpretable markers of disease (Honey and Sporns, 2008; Alstott et al., 2009; de Haan et al., 2012; Yang et al., 2016; Zimmermann et al., 2018; Singh et al., 2020). In this brief review we discuss different types of computational models at whole-brain and network level. We will explore their ability to recapitulate important aspects of empirical neural recordings, and end with new advances aimed at disease contexts and in the study of dynamic brain activity and behavior.

The contribution of this paper is threefold. First, it summarizes computational approaches that seek to understand how rich spatial and temporal structures can arise in brain activity using a set of underlying biophysical processes and their parameters. Second, it highlights several ways in which computational models are able to recapitulate empirical data across relevant scales ranging from electrophysiological scales corresponding to M/EEG signals to hemodynamic scales corresponding to fMRI signals. Our focus here is on whole brain network models of brain activity, rather than neuron or local circuit models. For readers interested in fine-scale or multi-scale modeling, we refer to a recent review (Glomb et al., 2021). Third, it discusses recent advances that attempt to make these complex computational models relevant to clinical and practical applications, often by deliberately simplifying these models and only keeping the most essential elements. The scope of the review is more focused on biophysical models rather than statistical graph theoretic metrics of SC-FC relationships. The field is greatly expanding and contains promising advances both from research and clinical standpoints. We believe that this article will serve to inform a wide readership about recent modeling work that are important from a practical point of view, yet are at risk of being inaccessible to a wide audience due to their mathematical or computational nature.

### 1.1. Magneto- and electroencephalography

We begin our report with a quick introduction to MEG and its older cousin EEG. These are non-invasive neuroimaging techniques that measure the postsynaptic potentials of pyramidal neurons in the brain (Biasiucci et al., 2019) by electrodes placed on the scalp (da Silva, 2013; Tivadar and Murray, 2019) or sensitive magnetic field detectors placed on or near the scalp (Hämäläinen et al., 1993; Gross et al., 2021). Both MEG and EEG possess high temporal resolution in the millisecond timescale, with spatial resolution determined by algorithms that solve the electromagnetic inverse problem (Biasiucci et al., 2019; Tivadar and Murray, 2019). The propagation of the signal from deep brain tissue to the scalp takes place due to the conductive properties of brain and head tissues, a phenomenon known as volume conduction which is also referred to as the electromagnetic forward problem (Kajikawa and Schroeder, 2011). To solve the inverse problem, source reconstruction algorithms (Michel et al., 2004) have been developed to estimate neural activity originating in deeper tissue, from the sensor signals and then superposing estimated functional activity on structural brain MRI, giving voxel-level signals. These algorithms have turned M/EEG into brain imaging technologies. However, it should be noted that source reconstruction is a mathematical ill-posed inverse problem; the reliability and spatial resolution of reconstructed signals are often functions of properties of the forward model as well as the signal and measurement noise. Once brain-wide neural sources and their activity have been reconstructed, further analysis might proceed analogously to imaging data like fMRI. The relationship between the functional activity from MEG/EEG/fMRI and the SC have been explored using various mathematical models, which we summarize in the next section.

## 2. Mathematical models

### 2.1. Coupled or networked non-linear neural mass models (NMMs)

At the mesoscopic scale, a lumped version of neural field was first described by Wilson and Cowan (1973); Cowan et al. (2016), consisting of an inhibitory and an excitatory population. Variants of this model were proposed by Da Silva et al. (1974) and Jansen and Rit (1995). Neural mass and neural field models are able to reproduce a range of dynamical behaviors that are observed in M/EEG, like oscillations in typical frequency bands (David and Friston, 2003), phase-amplitude-coupling (Onslow et al., 2014; Sotero, 2016), and evoked responses (Jansen et al., 1993; Jansen and Rit, 1995; David et al., 2005). Frequency-domain NMMs have also been proposed in order to model the M/EEG power spectrum (David and Friston, 2003; Bojak and Liley, 2005; Moran et al., 2007; Razi and Friston, 2016; Pereira et al., 2021).

By coupling together more than one lumped neural mass, one can start investigating the effect that inter-regional or remote connectivity and the resultant delays have on neural activity (Jirsa and Haken, 1996). Almost three decades ago, Jansen and Rit (1995) had already coupled together two neural mass models in order to simulate the effect of interactions between cortical columns on their activity. The Wilson-Cowan single oscillator model (Wilson and Cowan, 1972) too has evolved into a family of macroscopic coupled NMMs, with extensions to neocortical dynamics (Cowan et al., 2016; Deco et al., 2019b), controllability of brain networks (Muldoon et al., 2016), biomarkers in disease (Zimmermann et al., 2018), and second order statistics of observed brain signals (Deco et al., 2009; Abeysuriya et al., 2018; Wang et al., 2019; Byrne et al., 2020; Singh et al., 2020).

It is also common to use chaotic oscillators with little biophysical relevance (e.g., Yeung and Strogatz, 1999; Kuramoto, 2003), as the local neural mass oscillating around a given natural frequency (e.g., gamma band). Although there is no direct biological counterpart to these artificial models, they are sufficient to produce realistic synchrony and correlation properties (Deco et al., 2009; Cabral et al., 2014), e.g., the Kuramoto order parameter was linked to how dispersed firing is within a population (Byrne et al., 2017, 2020). Node-level Kuramoto oscillators were coupled using realistic brain connectivity and time delays determined by fiber lengths (Cabral et al., 2014; Finger et al., 2016).

Coupled NMMs are rapidly moving from mathematical curiosities to real applications in healthy and diseased brains (Honey and Sporns, 2008; Alstott et al., 2009; de Haan et al., 2012; Yang et al., 2016; Zimmermann et al., 2018; Singh et al., 2020) and can potentially be used to investigate biological processes such as neuromodulation (Shine et al., 2018, 2021; John et al., 2022). Such NMMs have also been simulated *via* the Virtual Brain platform—an open source neuroinformatics tool that provides access to brain network simulation tools (Ritter et al., 2013; Sanz-Leon et al., 2015). This tool has been used to study various diseases such as tumor (Aerts et al., 2018), Alzheimer's disease (Zimmermann et al., 2018), stroke (Falcon et al., 2015), and epilepsy (Jirsa et al., 2017).

Over the past few years, a vast literature has been developed on coupled neural models in a framework called dynamic causal modeling (DCM) (Kiebel et al., 2008; Pinotsis et al., 2012). DCM is a Bayesian framework for estimating directional interactions between coupled brain regions, i.e., the effective connectivity. A key concept underlying DCM is to treat the brain as a non-linear dynamic system that accepts multiple inputs and produces multiple outputs (i.e., MIMO model). This neuronal MIMO model is augmented with a regionally specific forward model that describes the mapping from neuronal activity to observations (MEG, EEG, local field potential; LFP, or fMRI). The neuronal and observation model together comprise a generative model that is subject to Bayesian inference. Stochastic DCMs are the most common generative models that have been used to test competing hypotheses about networks comprising only a few regions. Stochastic DCMs have usually proved to be highly successful at inferences of small networks (<10 nodes) than at large networks spanning the entire brain. However, increasing the number of regions or nodes in a stochastic DCM results in models with an enormous number of free parameters and profound conditional dependencies among the parameters, with exponential increases in computational time. Approaches such as spectral domain DCMs have been proposed to address this prohibitive estimation problem (Pereira et al., 2021), but there have been few attempts at whole-brain structural connectome integration with DCMs for M/EEG data.

### 2.2. Statistical models and graph metrics analysis

If the goal of structure-function modeling is merely to reproduce the second order covariances of neural activity, it is not necessary to employ a detailed biophysical signal generation model. Many recent studies have therefore focused on low-dimensional processes involving diffusion or random walks on the structural graph instead of mean-field models, under the assumption that such walks or network-diffusion processes can roughly simulate functional connectivity. These class of models were able to match or exceed the predictive power of complex coupled NMMs in predicting empirical FC (Abdelnour et al., 2014). Higher-order walks on graphs have also been quite successful; typically these methods involve a series expansion of the graph adjacency or Laplacian matrices (Meier et al., 2016; Becker et al., 2018). The diffusion and series expansion methods are themselves closely related (Robinson et al., 2016; Deslauriers-Gauthier et al., 2020; Tewarie et al., 2020), and naturally employ the so-called eigenmodes, or harmonics, of graph adjacency or Laplacian matrix. In these models, the eigenvectors of SC and FC are shared, and the functional eigenvalues are related by an exponentially-decaying function of the structural Laplacian eigenvalues. Hence these methods were generalized to yield spectral graph models whereby e.g., Laplacian harmonics were sufficient to reproduce empirical FC, using only a few eigenmodes (Atasoy et al., 2016; Abdelnour et al., 2018; Preti and Van De Ville, 2019). Although these models were initially demonstrated on fMRI-based FC, they have been readily demonstrated on M/EEG (Meier et al., 2016; Tewarie et al., 2019). Such spectral graph models are computationally attractive due to low-dimensionality and more interpretable analytical solutions. The SC's Laplacian eigenmodes may be thought of as the substrate on which functional patterns of the brain are established *via* a process of network transmission (Atasoy et al., 2016; Robinson et al., 2016; Abdelnour et al., 2018; Preti and Van De Ville, 2019; Glomb et al., 2020). Another class of models, the linear time-invariant system models based on the SC, have also been widely used to estimate brain controllability and energy required to transition to different states (Gu et al., 2015, 2017; Tang and Bassett, 2018; Stiso et al., 2019; Srivastava et al., 2022).

## 3. Challenges in inference of non-linear simulation models

The impressive richness and dynamic repertoire manifested by the above coupled neural mass and field models comes at a very high cost: that of model inference. These models are often mathematically complex (non-linearities), computationally complex (too many parameters) and statistically complex (ill-posed inference, non-unique solutions, broadly distributed posteriors). In addition, since they require long-time simulations involving numerical integration of highly coupled ODEs, they tend to be computationally costly, taking inordinately long time and computer power to create simulations that cover the entire parameter space.

The critical factor underlying these challenges is the coupling between a large number of non-linear NMMs. The complexity of coupled non-linear systems accommodates chaotic behavior which certainly imparts these models with a rich dynamic repertoire. Typically, coupled non-linear systems are characterized by exhibit discontinuous and abrupt shifts to oscillating, unstable, or chaotic behavior when network coupling or external driving force push the system over a bifurcation point. This produces a non-convex cost function with many local minima with discontinuous switching between regimes and model behavior—all adding up to highly challenging parameter inference. Recently, these challenges have been addressed using a two-step optimization procedure. First, all biophysical parameters of the local NMMs are set manually, without the use of empirical data, to be near an appropriate Hopf bifurcation point that gives the correct frequency (e.g., alpha band) (Sanz-Leon et al., 2015; Breakspear, 2017). In the second stage, optimization of the remaining few (global) parameters are performed by fitting to empirical FC metrics such as pairwise correlation or synchrony (Deco et al., 2009; Honey et al., 2009; Abeysuriya et al., 2018; Zimmermann et al., 2018; Demirtas et al., 2019; Wang et al., 2019). Even this optimization problem is usually no, zimmermann2018differentiationn-convex, hence grid search is employed instead of more sophisticated sampling methods. It is noteworthy that this two-step fitting procedure typically does not attempt to fit directly to actual time series of individual brain regions, only their second order FC, or in some cases the switching in time of granular “brain states” (Hansen et al., 2015; Deco et al., 2019a).

Two recent studies provide a thoughtful analysis of these inference problems. Hartoyo et al. (2019) highlighted the problem of unidentifiabiliy in whole brain models, where different parameter combinations can generate similar model predictions. Out of the 22 unknown parameters of a linearized network model they implemented, only one parameter was found to be identifiable when fitted to EEG data. Xie et al. (2019) show, using the Wilson-Cowan system as an archetype, that a large system of coupled NMMs may be pre-specified for firing rates pertaining to a specified frequency, but extending such a model to the network scale does not allow accurate, reliable, or efficient optimization. In particular, the manual/grid search fitting described above does not then also predict regional signals' power spectra. It was rightly noted in a recent review that coupled NMMs do not necessarily aim at maximizing the fit to the empirical brain signals but to reproduce specific temporal, spatial, or spectral features of the empirical data emerging at the macroscopic scale whose underlying mechanisms remain unclear (Glomb et al., 2021).

Due to these challenges, to date, coupled NMMs have not found widespread or routine usage in clinical or translational settings. One way to make the leap to practice is to carefully assess which modeling aspects may be considered useful, and which may be discarded. For instance, if the goal is to reproduce covariances rather than the signal spectra, then is a full-strength coupled NMM even necessary?

## 4. Recent advances in structure-function models

Below we highlight a few recent attempts that seek to enhance the practical applicability of complex computational models. Often these attempts have taken a step back from the complexity of current models and have instead explored deliberate simplifications rather than enhancements to the theoretical models. For example, many of the following coupled NMMs attempts involve fixing the local neural mass model parameters and then tuning only a small set of global parameters, simplify them to consist of fewer parameters, or linearize local masses to allow better inference.

### 4.1. Advances in coupled NMMs

In one study, the non-linear dynamics of coupled NMMs was transformed by applying a Lotka-Volterra type transform (Galadi et al., 2021). This enabled tracking of the temporal evolution of the global attractor and the non-stationary attractor landscape. Using this approach, the authors were able to compare awake state with different stages of sleep with BOLD fMRI data (Galadi et al., 2021). A recent study modeled noisy Stuart-Landau oscillators to simulate BOLD fMRI and found that low-level states of consciousness were associated with structural hub regions losing stability and an increase in homogeneity in the brain regional dynamics (López-González et al., 2021). Another study on the levels of consciousness demonstrated that in addition to the descriptive distance metrics classifying the different levels of consciousness, the extent of perturbation required to switch between the levels provide complementary information about the levels. Here, the extent of perturbation was quantified by introducing a perturbation term in the coupled NMMs (Sanz Perl et al., 2021). A DCM approach was used to investigate modulation of gain by the cognitive control systems in early psychosis (Burgher et al., 2021).

In addition to exploring brain states, a recent study demonstrated how NMMs can be enriched by incorporating brain-wide transcriptional data that captures the regional inhibitory and excitatory receptor gene expression. This approach ensures model parsimony while incorporating regional heterogeneity to simulate rich BOLD fMRI dynamics (Deco et al., 2021). While these studies explored biological and clinical applications of the NMMs, a recent study also showed how NMMs can be leveraged to infer virtual FC or SC. The inferred virtual connectomes's classification of healthy vs. AD as well as different age classes was comparable to the classification done by the empirical connectomes (Arbabyazd et al., 2021). Lastly, a next generation NMM was employed to investigate the role of structural connectivity in regulating the emergent functional dynamics (Gerster et al., 2021).

Recent advances were also made using the Virtual Brain platform. One of the studies demonstrated selectively stimulating different fiber tracts in a personalized virtual brain model to simulate network effects in deep brain stimulation for patients with treatment-resistant depression (An et al., 2022). Another application of the virtual brain platform was to demonstrate similarity between neuronal cascades of firing rate fluctuations and resting state networks dynamics (Rabuffo et al., 2021).

Coupled NMMs have also been leveraged to fit to M/EEG. Recently, coupled phase oscillator models was used to reconstruct coupling between frequency bands (interlayer connectivity) obtained from MEG (Tewarie et al., 2021). Another study focused on estimating coupled NMMs from EEG recordings of epilepsy patients to develop a closed-loop deep brain stimulator (Chang et al., 2021).

### 4.2. Advances in statistical models and graph metrics analysis

**Figure 1:**
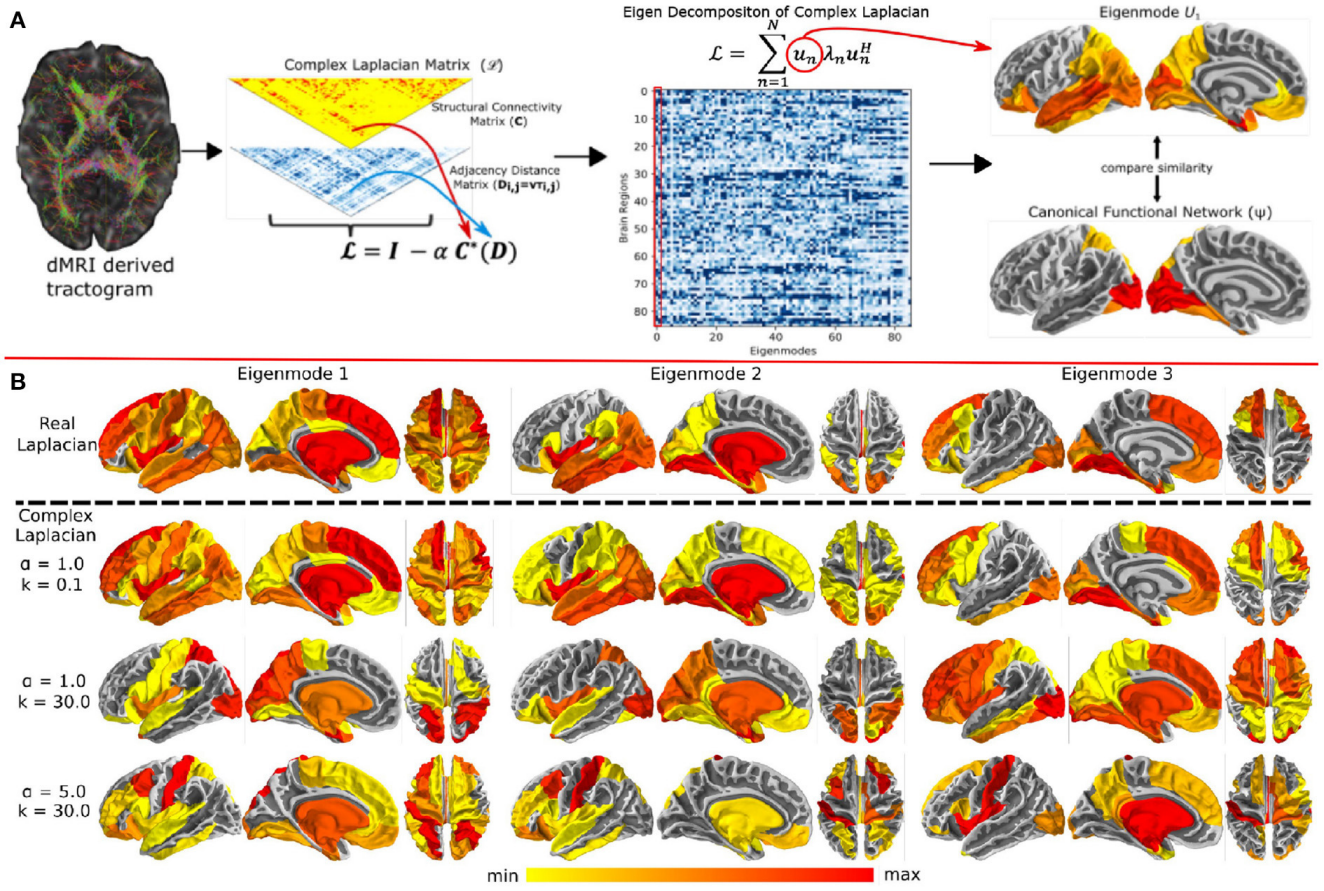
**(A)** Overview of the complex Laplacian eigenmode decomposition. The complex Laplacian matrix L is computed from the structural connectivity matrix (**C**) and distance adjacency matrix (**D**) which were extracted from diffusion MRI. Then, eigendecomposition of the Laplacian L provided the complex structural eigenmodes. The spatial similarities between these eigenmodes and the canonical functional networks in fMRI was done for model parameter tuning. **(B)** Complex Laplacian eigenmodes for different coupling strength and wave number. Top row: eigenmodes from the real Laplacian matrix with coupling strength = 1. Bottom three rows: Complex Laplacian eigenmodes for small wave number (top), high wave number (middle), and high wave number and coupling strength (bottom). Figure is extracted from Xie et al. (2021).

To improve the structure-function model fits, a new proposal relates the eigenvalues of the structural connectivity and functional networks using the Gamma function instead of the exponential decay, producing a reliable prediction of functional connectivity with a single model parameter (Cummings et al., 2022). They also found that adding small levels of long range interhemispheric connectivity greatly improves model performance, since it is well-known that DTI-based fiber connectivity methods significantly under-estimate inter-hemispheric connections. Another recent study extended the notion of graph Laplacian by introducing global connectome coupling and a conductance speed parameter, which gave rise to a “tunable” Laplacian (Xie et al., 2021) (Figure 1A). Since delays becomes phases in Fourier space, this study proposed a so-called “complex Laplacian”. They showed that with the right selection of the two model parameters, it is possible to “steer” the (complex) eigenmodes of this Laplacian in such a manner that a small number of them can reproduce any given canonical functional network (like default mode, salience, etc.) at relevant frequencies. Examples of these complex eigenmodes were shown in Figure 1B, copied from the original publication (Xie et al., 2021). This is exciting, since it can accommodate a large repertoire of microstates and their concomitant spatial patterns—an essential characteristic of real brain activity. This rich repertoire could be engaged using only two steerable model parameters, suggesting the possibility that complex behavior may be achievable by simple and parsimonious mechanisms. A latest study compared the performance of 40 structure-function coupling models in capturing the variance in FC (Zamani Esfahlani et al., 2022). They found that having regional heterogeneous coupling is more accurate than having a global coupling between structure and function. Moreover, specific models were more accurate in capturing the coupling between specific brain regions. Lastly, this coupling decreased with age. Another study showed a stronger association of structural eigenmodes (in comparison to structural connections) with phase and amplitude connectivity of MEG at short time-scales (Tewarie et al., 2022).

Network control models have also been recently extended and applied to investigate controllability in drug-resistant focal epilepsy in children (Chari et al., 2022), functional dynamics mediated by controllability of the structural connectivity (Gu et al., 2022), prediction of aphasia recovery using controllability measures (Wilmskoetter et al., 2022), association of controllability measures with normative negative affect variability (McGowan et al., 2021), prediction of positive psychosis spectrum symptoms with controllability measures (Parkes et al., 2021), role of controllability measures in language tasks (Medaglia et al., 2021), altered energy landscape and stability of working memory representations in schizophrenia (Braun et al., 2021), structural control energy in psychosis vulnerability (Zoller et al., 2021), and dynamics of controllability measures during seizure progression (Scheid et al., 2021).

### 4.3. Spectral graph model for neural oscillations

Above-noted spectral graph models were successful in capturing steady-state, stationary second order covariances in brain activity, but did not possess oscillatory behavior. Hence their extension to accommodate a larger repertoire of dynamically-varying microstates or rich power spectra at higher frequencies typically observed on EEG or MEG would require a full accounting of axonal propagation delays as well as local neural population dynamics within graph models, as previously advocated (Cabral et al., 2011). Band-specific MEG resting-state networks were successfully modeled with a combination of delayed neural mass models and eigenmodes of the structural network (Tewarie et al., 2019), suggesting delayed interactions in a brain's network give rise to functional patterns constrained by structural eigenmodes.

Recently another effort was undertaken to characterize wide-band brain activity using graph harmonics in closed form (i.e., requiring no time-domain simulations), a rarity in the field of computational neuroscience (Raj et al., 2020). This spectral graph model (SGM) of wideband brain activity produced realistic power spectra that could successfully predict both the spatial as well as temporal properties of MEG empirical recordings (Raj et al., 2020). Remarkably, the model has very few (seven) parameters, all of which are global and not dependent on local oscillations. This method therefore exemplifies the power of graph methods in reproducing more complex and rich repertoire of brain activity, while keeping to a parsimonious approach that skirts the need for high-dimensional and non-linear oscillatory NMMs. SGM can be interpreted as a linearized coupled NMM.

**Figure 2:**
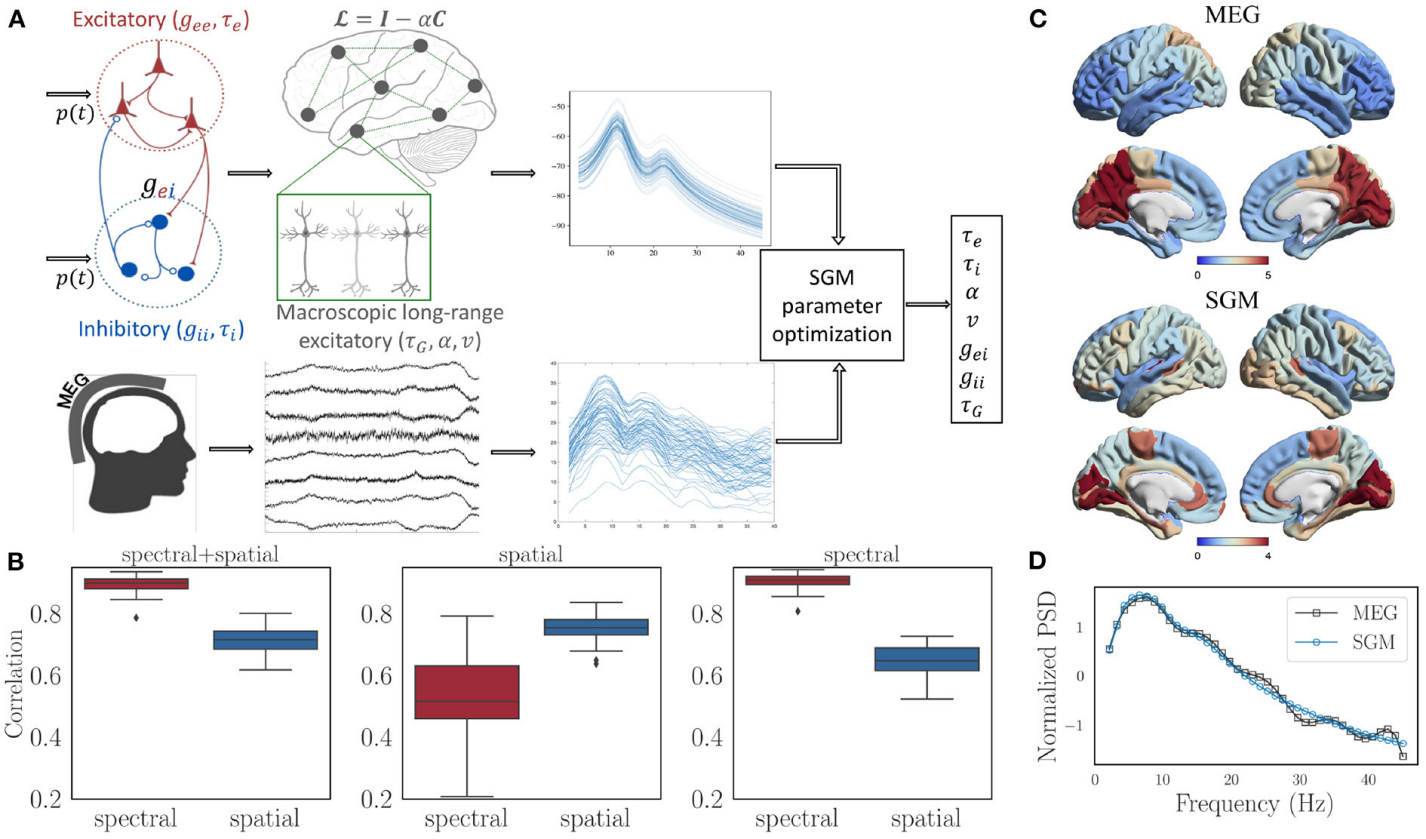
**(A)** Flow chart showing the structure of SGM and inference of SGM parameters by comparing modeled frequency spectra with the empirical frequency spectra captured by MEG. Input *p*(*t*) is white Gaussian noise and is the same for both excitatory as well as inhibitory signals. **(B)** Spectral and spatial correlations obtained after optimization for all the subjects with three different cost functions: (1) spectral + spatial correlation, 2) spatial correlation, 3) spectral correlation. **(C)** Comparison of the empirical and modeled spatial distribution of the alpha frequency band, averaged over all the subjects. **(D)** Comparison of empirical and modeled frequency spectra, averaged over all brain regions and subjects.

The SGM consists of two levels. At the mesoscopic (local or node) level the excitatory and inhibitory neuronal populations are characterized by time constants τ_e_, τ_*I*_ and gains *g*_ee_, *g*_ei_, and *g*_ii_. At the macroscopic level long-range pyramidal excitatory neuronal populations are characterized by time constant τ_G_, and a coupling constant α. A pictorial depiction of this model is shown in Figure 2A. Remarkably, the SGM is available in closed-form in Fourier space as a summation over the eigenmodes of the complex Laplacian L(ω):


(1) $$X(\omega )=\overset{N}{\underset{k=1}{\sum^{\text{​}}}}\frac{u_{k}(\omega )u_{k}{\left(\omega \right)}^{\text{H}}}{\text{j}\omega +{\text{τ}}_{\text{G}}^{-1}{\text{λ}}_{k}(\omega )F_{\text{G}}(\omega )}H_{\text{local}}(\omega )P(\omega ),$$

where ω is the angular frequency, **u**_*k*_(ω) are the eigenmodes and λ_*k*_(ω) are the eigenvalues. The term *H*_local_(ω) refer to the mesoscopic model's transfer function, *P*(ω) to the input noise spectrum, and *F*_G_(ω) is the neural impulse response transfer function (Gamma shaped). Details and derivation of this equation from first principles are contained in Raj et al. (2020) and Verma et al. (2022a). The structure of SGM is similar to that of Jansen-Rit model (Jansen and Rit, 1995; Sanz-Leon et al., 2015). Further methodological comparisons can be found elsewhere (Verma et al., 2022b).

Since the development of the SGM, there have been various extensions as well. We recently improved SGM by enhancing the biophysical interpretability of the mesoscopic model (Verma et al., 2022a), and subsequently demonstrated various stability properties of this improved model (Verma et al., 2022b). In the latter, we also outlined a strategy to capture temporal fluctuations in MEG by fitting the SGM parameters to MEG frequency spectra at various time points. The mesoscopic SGM model parameters were also estimated to differentiate Alzheimer's disease with healthy controls (Ranasinghe et al., 2022), as well as different stages of sleep (Fan et al., 2022).

### 4.4. Parameter inference of SGM from empirical MEG Data

The key benefit of linear graph models like the SGM over coupled NMMs is that parameter inference is far simpler and easily accomplished in a data-driven fashion without the need for manual selection. While prior works showed that simple simulated annealing-type optimization can accomplish this task, there is still an open question regarding the exact form of the cost function and which data features to use for inference. Here we highlight very recent developments in this area. Broadly, prior NMM methods have focused on FC as the target of optimization, whereas prior SGM studies used wideband MEG spectra directly. Here we show that SGM is capable of fitting simultaneously to both spectra and spatial distributions of the alpha frequency band.

We used the same MEG dataset reported previously (Xie et al., 2020; Verma et al., 2022a), comprising of 36 healthy adult subjects (23 males, 13 females; 26 left-handed, 10 right-handed; mean age 21.75 years, age range 7–51 years), having 1 min MEG recordings at rest and eyes closed. SGM was computed on the structural connectomes of these subjects, and both modeled and empirical MEG were converted into power spectral densities (PSD) in dB scale. Pearson's *r* between modeled and MEG PSD was computed for all 68 brain regions. Its average *r* across all regions is referred to as the *spectral correlation*. Next we calculated the *spatial correlation* by obtaining the regional distribution of alpha band (8-12 Hz) raw power of both model **x** and MEG **y**. Then, the spatial correlation was defined as **x**^*T*^ǁ(**C**+*w***I**)ǁ**y**, where **C** is the row degree normalized structural connectivity matrix, **I** is the identity matrix, *w* is an empirical weight, and ǁ(**C**+*w***I**)ǁ is the row normalized version of **C**+*w***I**. The objective function for optimization and estimation of model parameters was the sum of spectral and spatial correlations. The details of the optimization procedure are in the Supplementary material.

#### 4.4.1. Model parameter estimation results

We firstly tested different values of *w* in the spatial correlation weight of *C*+w*I. The values being tested were 0.01, 0.1, 0, 1, and 10. For each of the values of *w*, no statistically different spectral correlations were observed based on a Tuckey's HSD test on Fisher's r-to-z transformed correlations. However, the spatial correlations were highest for *w* = 10. Therefore, we finally chose a value of *w* = 10 out of 0.01, 0.1, 0, 1, and 10 for subsequent analysis. After performing model parameter optimization for all the subjects with *w* fixed at 10, we see that the spectral as well as spatial correlations are high for all of them, as shown in the left-most subplot in Figure 2B. In addition, the spectral correlation reduces significantly when spatial correlation alone is used as a cost function for optimization (middle subplot in Figure 2B), and the spatial correlation reduces significantly when spectral correlation alone is used as a cost function (right-most subplot in Figure 2B). Therefore, we used an unweighted sum of spectral and spatial correlations for final analysis. Visually, we see an accurate match for the spatial distribution of the alpha band (Figure 2C) as well as the spectral shape (Figure 2D).

This represents a noteworthy advance over prior publications, since the inferred model is informed equally by spectral as well as spatial features of empirical MEG—unlike most comparable methods described in this paper. We believe future computational approaches may similarly benefit from the use of multiple feature classes of empirical functional data.

In addition to the approach above, NMMs have also been linearized previously. Such linearized NMMs, both at a regional level and for the entire brain network (Moran et al., 2007; Deco et al., 2014; Abeysuriya and Robinson, 2016; Gabay et al., 2018; Raj et al., 2020; Verma et al., 2022a) provide for tractable parameter inference in comparison to their non-linear counterparts. However, this simplification limits the repertoire of dynamical solutions that these models can exhibit, similar to SGM.

## 5. Conclusions

We briefly surveyed the burgeoning field of computational neural mass models on the whole brain connectivity network. Such models have been widely used to infer the underlying biophysics of the whole brain activity from non-invasive neuroimaging data. They have been used to study various neurological disease as well as brain states.

We noted that most current methods were designed to achieve resemblance to functional connectivity—a second-order feature based on measuring co-activity between brain regions—rather than to the data itself. Although this is justified in specific use-cases, we argued that it would be necessary to develop more complete models that can achieve both goals. We noted potential scientific and clinical impact of current approaches, but enumerated several challenges associated with model inference. In summary, the challenges are: (i) parameter identifiability issues when model parameters are heterogeneous across brain regions, (ii) parameter inference difficulty due to abrupt shift in model behavior with small shifts in parameters, and (iii) fitting directly to actual time series instead of second order FC.

We identified many recent and exciting advances, in both non-linear coupled NMM approaches, as well as the recent emergence of linearized spectral graph models. The latter in particular present an attractive trade-off between model complexity, accuracy and practicality. We conclude that while the field has seen various advancements with applications, further advances are needed to overcome identified issues. Therefore in this review, we suggest the following directions for further advancement of this field:

1. Develop computational models that can capture temporal, spatial, as well as spectral features of whole brain imaging.
2. Develop parsimonious biophysical computational models that can be used in clinical or translational settings.

We are excited by the recent highlighted advances, that have together taken meaningful steps in this direction.

## Author contributions

AR, PV, and SN conceptualized and designed the SGM results. AR and SN supervised the SGM results. PV generated and compiled the SGM results. AR created the first draft. All authors contributed to the writing and editing of the manuscript. All authors contributed to the article and approved the submitted version.

## Funding

This work was partially supported by NIH grants R01AG072753, R01NS092802, R01NS018341, R01EB022717, R01DC013979, R01NS100440, R01DC176960, R01DC017091, R01AG062196, UCOP-MRP-17-454755, DOD CDMRP Grant W81XWH1810741, Alzheimer's Association Grant AARFD-22-923931, and an industry research contract from Ricoh MEG Inc.

## Conflict of interest

The authors declare that the research was conducted in the absence of any commercial or financial relationships that could be construed as a potential conflict of interest.

## Publisher's note

All claims expressed in this article are solely those of the authors and do not necessarily represent those of their affiliated organizations, or those of the publisher, the editors and the reviewers. Any product that may be evaluated in this article, or claim that may be made by its manufacturer, is not guaranteed or endorsed by the publisher.

## References

[B1] Abdelnour F. Dayan M. Devinsky O. Thesen T. Raj A. (2018). Functional brain connectivity is predictable from anatomic network's Laplacian eigen-structure. Neuroimage 172, 728–739. 10.1016/j.neuroimage.2018.02.016 29454104 PMC6170160

[B2] Abdelnour F. Voss H. U. Raj A. (2014). Network diffusion accurately models the relationship between structural and functional brain connectivity networks. Neuroimage 90, 335–347. 10.1016/j.neuroimage.2013.12.039 24384152 PMC3951650

[B3] Abeysuriya R. Robinson P. (2016). Real-time automated EEG tracking of brain states using neural field theory. J. Neurosci. Methods 258, 28–45. 10.1016/j.jneumeth.2015.09.026 26523766

[B4] Abeysuriya R. G. Hadida J. Sotiropoulos S. N. Jbabdi S. Becker R. Hunt B. A. E.. (2018). A biophysical model of dynamic balancing of excitation and inhibition in fast oscillatory large-scale networks. PLoS Comput. Biol. 14, e1006007. 10.1371/journal.pcbi.1006007 29474352 PMC5841816

[B5] Aerts H. Schirner M. Jeurissen B. Van Roost D. Achten E. Ritter P.. (2018). Modeling brain dynamics in brain tumor patients using the virtual brain. eNeuro 5. 10.1523/ENEURO.0083-18.2018 29911173 PMC6001263

[B6] Alstott J. Breakspear M. Hagmann P. Cammoun L. Sporns O. (2009). Modeling the impact of lesions in the human brain. PLoS Comput. Biol. 5, e1000408. 10.1371/journal.pcbi.1000408 19521503 PMC2688028

[B7] An S. Fousek J. Kiss Z. H. Cortese F. van der Wijk G. McAusland L. B.. (2022). High-resolution virtual brain modeling personalizes deep brain stimulation for treatment-resistant depression: Spatiotemporal response characteristics following stimulation of neural fiber pathways. Neuroimage 249, 118848. 10.1016/j.neuroimage.2021.118848 34954330

[B8] Arbabyazd L. Shen K. Wang Z. Hofmann-Apitius M. Ritter P. McIntosh A. R.. (2021). Virtual connectomic datasets in Alzheimer's disease and aging using whole-brain network dynamics modelling. eNeuro 8. 10.1523/ENEURO.0475-20.2021 PMC8260273 34045210

[B9] Atasoy S. Donnelly I. Pearson J. (2016). Human brain networks function in connectome-specific harmonic waves. Nat. Commun. 7, 10340. 10.1038/ncomms10340 26792267 PMC4735826

[B10] Bassett D. S. Bullmore E. T. (2009). Human brain networks in health and disease. Curr. Opin. Neurol. 22, 340. 10.1097/WCO.0b013e32832d93dd 19494774 PMC2902726

[B11] Becker C. Pequito S. Pappas G. Miller M. Grafton S. Bassett D.. (2018). Spectral mapping of brain functional connectivity from diffusion imaging. Nat. Sci. Rep. 8, 1–15. 10.1038/s41598-017-18769-x 29362436 PMC5780460

[B12] Biasiucci A. Franceschiello B. Murray M. M. (2019). Electroencephalography. Curr. Biol. 29, R80?R85. 10.1016/j.cub.2018.11.052 30721678

[B13] Bojak I. Liley D. (2005). Modeling the effects of anesthesia on the electroencephalogram. Phys. Rev. E 71, 041902. 10.1103/PhysRevE.71.041902 15903696

[B14] Braun U. Harneit A. Pergola G. Menara T. Schäfer A. Betzel R. F.. (2021). Brain network dynamics during working memory are modulated by dopamine and diminished in schizophrenia. Nat. Commun. 12, 1–11. 10.1038/s41467-021-23694-9 34108456 PMC8190281

[B15] Breakspear M. (2017). Dynamic models of large-scale brain activity. Nat. Neurosci. 20, 340–352. 10.1038/nn.4497 28230845

[B16] Burgher B. Whybird G. Koussis N. Scott J. G. Cocchi L. Breakspear M. (2021). Sub-optimal modulation of gain by the cognitive control system in young adults with early psychosis. Transl. Psychiatry 11, 1–9. 10.1038/s41398-021-01673-4 34707092 PMC8551269

[B17] Byrne A. Brookes M. J. Coombes S. (2017). A mean field model for movement induced changes in the beta rhythm. J. Comput. Neurosci. 43, 143–158. 10.1007/s10827-017-0655-7 28748303 PMC5585324

[B18] Byrne Á. O'Dea R. D. Forrester M. Ross J. Coombes S. (2020). Next-generation neural mass and field modeling. J. Neurophysiol. 123, 726–742. 10.1152/jn.00406.2019 31774370

[B19] Cabral J. Hugues E. Sporns O. Deco G. (2011). Role of local network oscillations in resting-state functional connectivity. Neuroimage 57, 130–139. 10.1016/j.neuroimage.2011.04.010 21511044

[B20] Cabral J. Kringelbach M. L. Deco G. (2014). Exploring the network dynamics underlying brain activity during rest. Prog. Neurobiol. 114, 102–131. 10.1016/j.pneurobio.2013.12.005 24389385

[B21] Cao M. Wang J.-H. Dai Z.-J. Cao X.-Y. Jiang L.-L. Fan F.-M.. (2014). Topological organization of the human brain functional connectome across the lifespan. Dev. Cogn. Neurosci. 7, 76–93. 10.1016/j.dcn.2013.11.004 24333927 PMC6987957

[B22] Chang S. Wang J. Liu C. Yi G. Lu M. Che Y.. (2021). A data driven experimental system for individualized brain stimulation design and validation. IEEE Trans. Neural Syst. Rehabil. Eng. 29, 1848–1857. 10.1109/TNSRE.2021.3110275 34478377

[B23] Chari A. Seunarine K. K. He X. Tisdall M. M. Clark C. A. Bassett D. S.. (2022). Drug-resistant focal epilepsy in children is associated with increased modal controllability of the whole brain and epileptogenic regions. Commun. Biol. 5, 1–14. 10.1038/s42003-022-03342-8 35484213 PMC9050895

[B24] Cowan J. D. Neuman J. van Drongelen W. (2016). Wilson-Cowan equations for neocortical dynamics. J. Math. Neurosci. 6, 1–24. 10.1186/s13408-015-0034-5 26728012 PMC4733815

[B25] Cummings J. A. Sipes B. Mathalon D. H. Raj A. (2022). Predicting functional connectivity from observed and latent structural connectivity via eigenvalue mapping. Front. Neurosci. 16, 810111. 10.3389/fnins.2022.810111 35368264 PMC8964629

[B26] da Silva F. L. (2013). EEG and MEG: relevance to neuroscience. Neuron 80, 1112–1128. 10.1016/j.neuron.2013.10.017 24314724

[B27] Da Silva F. L. Hoeks A. Smits H. Zetterberg L. (1974). Model of brain rhythmic activity. Kybernetik 15, 27–37. 10.1007/BF00270757 4853232

[B28] David O. Friston K. J. (2003). A neural mass model for MEG/EEG:: coupling and neuronal dynamics. Neuroimage 20, 1743–1755. 10.1016/j.neuroimage.2003.07.015 14642484

[B29] David O. Harrison L. Friston K. J. (2005). Modelling event-related responses in the brain. Neuroimage 25, 756–770. 10.1016/j.neuroimage.2004.12.030 15808977

[B30] de Haan W. Mott K. van Straaten E. C. W. Scheltens P. Stam C. J. (2012). Activity dependent degeneration explains hub vulnerability in Alzheimer's disease. PLoS Comput. Biol. 8, e1002582. 10.1371/journal.pcbi.1002582 22915996 PMC3420961

[B31] Deco G. Cruzat J. Cabral J. Tagliazucchi E. Laufs H. Logothetis N. K.. (2019a). Awakening: predicting external stimulation to force transitions between different brain states. Proc. Natl. Acad. Sci. U.S.A. 116, 18088–18097. 10.1073/pnas.1905534116 31427539 PMC6731634

[B32] Deco G. Cruzat J. Kringelbach M. L. (2019b). Brain songs framework used for discovering the relevant timescale of the human brain. Nat. Commun. 10, 1–13. 10.1038/s41467-018-08186-7 30718478 PMC6361902

[B33] Deco G. Jirsa V. McIntosh A. R. Sporns O. Kotter R. (2009). Key role of coupling, delay, and noise in resting brain fluctuations. Proc. Natl. Acad. Sci. U.S.A. 106, 10302–10307. 10.1073/pnas.0901831106 19497858 PMC2690605

[B34] Deco G. Kringelbach M. L. Arnatkeviciute A. Oldham S. Sabaroedin K. Rogasch N. C.. (2021). Dynamical consequences of regional heterogeneity in the brain's transcriptional landscape. Sci. Adv. 7, eabf4752. 10.1126/sciadv.abf4752 34261652 PMC8279501

[B35] Deco G. Ponce-Alvarez A. Hagmann P. Romani G. L. Mantini D. Corbetta M. (2014). How local excitation-inhibition ratio impacts the whole brain dynamics. J. Neurosci. 34, 7886–7898. 10.1523/JNEUROSCI.5068-13.2014 24899711 PMC4044249

[B36] Demirtas M. Burt J. B. Helmer M. Ji J. L. Adkinson B. D.. (2019). Hierarchical heterogeneity across human cortex shapes large-scale neural dynamics. Neuron 101, 1181.e13–1194.e13. 10.1016/j.neuron.2019.01.017 30744986 PMC6447428

[B37] Deslauriers-Gauthier S. Zucchelli M. Frigo M. Deriche R. (2020). A unified framework for multimodal structure-function mapping based on eigenmodes. Med. Image Anal. 66, 101799. 10.1016/j.media.2020.101799 32889301

[B38] Falcon M. I. Riley J. D. Jirsa V. McIntosh A. R. Shereen A. D. Chen E. E.. (2015). The virtual brain: modeling biological correlates of recovery after chronic stroke. Front. Neurol. 6, 228. 10.3389/fneur.2015.00228 26579071 PMC4629463

[B39] Fan J. M. Kudo K. Verma P. Ranasinghe K. G. Morise H. Findlay A. M.. (2022). Whole brain network analysis of neural synchrony and information flow during transition from wakefulness to light non-rapid eye movement sleep. bioRxiv. 10.1101/2022.03.09.483562 PMC10697405 37788939

[B40] Finger H. Bönstrup M. Cheng B. Messé A. Hilgetag C. Thomalla G.. (2016). Modeling of large-scale functional brain networks based on structural connectivity from dti: comparison with eeg derived phase coupling networks and evaluation of alternative methods along the modeling path. PLoS Comput. Biol. 12, e1005025. 10.1371/journal.pcbi.1005025 27504629 PMC4978387

[B41] Fornito A. Zalesky A. Breakspear M. (2015). The connectomics of brain disorders. Nat. Rev. Neurosci. 16, 159–172. 10.1038/nrn3901 25697159

[B42] Gabay N. C. Babaie-Janvier T. Robinson P. A. (2018). Dynamics of cortical activity eigenmodes including standing, traveling, and rotating waves. Phys. Rev. E 98, 042413. 10.1103/PhysRevE.98.042413

[B43] Galadi J. Silva Pereira S. Sanz Perl Y. Kringelbach M. Gayte I. Laufs H.. (2021). Capturing the non-stationarity of whole-brain dynamics underlying human brain states. Neuroimage 244, 118551. 10.1016/j.neuroimage.2021.118551 34506913

[B44] Gerster M. Taher H. koch A. Hlinka J. Guye M. Bartolomei F.. (2021). Patient-specific network connectivity combined with a next generation neural mass model to test clinical hypothesis of seizure propagation. Front. Syst. Neurosci. 15, 675272. 10.3389/fnsys.2021.675272 34539355 PMC8440880

[B45] Glomb K. Cabral J. Cattani A. Mazzoni A. Raj A. Franceschiello B. (2021). Computational models in electroencephalography. Brain Topogr. 35, 142–161. 10.1007/s10548-021-00828-2 33779888 PMC8813814

[B46] Glomb K. Queralt J. R. Pascucci D. Defferrard M. Tourbier S. Carboni M.. (2020). Connectome spectral analysis to track EEG task dynamics on a subsecond scale. Neuroimage 2020, 117137. 10.1016/j.neuroimage.2020.117137 32652217

[B47] Gross J. Junghöfer M. Wolters C. (2021). Bioelectromagnetism in human brain research: New applications, new questions. Neuroscientist. 10.1177/10738584211054742 PMC9902961 34873945

[B48] Gu S. Betzel R. F. Mattar M. G. Cieslak M. Delio P. R. Grafton S. T.. (2017). Optimal trajectories of brain state transitions. Neuroimage 148, 305–317. 10.1016/j.neuroimage.2017.01.003 28088484 PMC5489344

[B49] Gu S. Fotiadis P. Parkes L. Xia C. H. Gur R. C. Gur R. E.. (2022). Network controllability mediates the relationship between rigid structure and flexible dynamics. Netw. Neurosci. 6, 275–297. 10.1162/netn_a_00225 PMC9810281 36605890

[B50] Gu S. Pasqualetti F. Cieslak M. Telesford Q. K. Alfred B. Y. Kahn A. E.. (2015). Controllability of structural brain networks. Nat. Commun. 6, 1–10. 10.1038/ncomms9414 26423222 PMC4600713

[B51] Hagmann P. Cammoun L. Gigandet X. Meuli R. Honey C. J. Wedeen V. J.. (2008). Mapping the structural core of human cerebral cortex. PLoS Biol. 6, e159. 10.1371/journal.pbio.0060159 18597554 PMC2443193

[B52] Hämäläinen M. Hari R. Ilmoniemi R. J. Knuutila J. Lounasmaa O. V. (1993). Magnetoencephalography-theory, instrumentation, and applications to noninvasive studies of the working human brain. Rev. Modern Phys. 65, 413. 10.1103/RevModPhys.65.413

[B53] Hansen E. C. Battaglia D. Spiegler A. Deco G. Jirsa V. K. (2015). Functional connectivity dynamics: Modeling the switching behavior of the resting state. NeuroImage 105, 525–535. 10.1016/j.neuroimage.2014.11.001 25462790

[B54] Hartoyo A. Cadusch P. J. Liley D. T. J. Hicks D. G. (2019). Parameter estimation and identifiability in a neural population model for electro-cortical activity. PLoS Comput. Biol. 15, e1006694. 10.1371/journal.pcbi.1006694 31145724 PMC6542506

[B55] Honey C. J. Sporns O. (2008). Dynamical consequences of lesions in cortical networks. Human Brain Mapping 29, 802–809. 10.1002/hbm.20579 18438885 PMC6870962

[B56] Honey C. J. Sporns O. Cammoun L. Gigandet X. Thiran J. P. Meuli R.. (2009). Predicting human resting-state functional connectivity from structural connectivity. Proc. Natl. Acad. Sci. U.S.A. 106, 2035–2040. 10.1073/pnas.0811168106 19188601 PMC2634800

[B57] Honey C. J. Thivierge J.-P. Sporns O. (2010). Can structure predict function in the human brain? Neuroimage 52, 766–776. 10.1016/j.neuroimage.2010.01.071 20116438

[B58] Jansen B. H. Rit V. G. (1995). Electroencephalogram and visual evoked potential generation in a mathematical model of coupled cortical columns. Biol. Cybernet. 73, 357–366. 10.1007/BF00199471 7578475

[B59] Jansen B. H. Zouridakis G. Brandt M. E. (1993). A neurophysiologically-based mathematical model of flash visual evoked potentials. Biol. Cybernet. 68, 275–283. 10.1007/BF00224863 8452897

[B60] Jirsa V. Proix T. Perdikis D. Woodman M. Wang H. Gonzalez-Martinez J.. (2017). The virtual epileptic patient: individualized whole-brain models of epilepsy spread. Neuroimage 145, 377–388. 10.1016/j.neuroimage.2016.04.049 27477535

[B61] Jirsa V. K. Haken H. (1996). Field theory of electromagnetic brain activity. Phys. Rev. Lett. 77, 960. 10.1103/PhysRevLett.77.960 10062950

[B62] John Y. J. Sawyer K. S. Srinivasan K. Müller E. J. Munn B. R. Shine J. M. (2022). It's about time: Linking dynamical systems with human neuroimaging to understand the brain. Netw. Neurosci. 1–20. 10.1162/netn_a_00230 36875012 PMC9976648

[B63] Kajikawa Y. Schroeder C. E. (2011). How local is the local field potential? Neuron 72, 847–858. 10.1016/j.neuron.2011.09.029 22153379 PMC3240862

[B64] Kiebel S. J. Garrido M. I. Moran R. J. Friston K. J. (2008). Dynamic causal modelling for EEG and MEG. Cogn. Neurodyn. 2, 121. 10.1007/s11571-008-9038-0 19003479 PMC2427062

[B65] Kuramoto Y. (2003). Chemical Oscillations, Waves, and Turbulence. Courier Corporation.

[B66] López-González A. Panda R. Ponce-Alvarez A. Zamora-López G. Escrichs A. Martial C.. (2021). Loss of consciousness reduces the stability of brain hubs and the heterogeneity of brain dynamics. Commun. Biol. 4, 1–15. 10.1038/s42003-021-02537-9 34489535 PMC8421429

[B67] McGowan A. L. Parkes L. He X. Stanoi O. Kang Y. Lomax S.. (2021). Controllability of structural brain networks and the waxing and waning of negative affect in daily life. Biol. Psychiatry Glob. Open Sci. 10.1016/j.bpsgos.2021.11.008 PMC9616346 36324655

[B68] Medaglia J. D. Harvey D. Y. Kelkar A. S. Zimmerman J. P. Mass J. A. Bassett D. S.. (2021). Language tasks and the network control role of the left inferior frontal gyrus. eNeuro 8. 10.1523/ENEURO.0382-20.2021 PMC8431826 34244340

[B69] Meier J. Tewarie P. Hillebrand A. Douw L. van Dijk B. W. Stufflebeam S. M.. (2016). A mapping between structural and functional brain networks. Brain Connect. 6, 298–311. 10.1089/brain.2015.0408 26860437 PMC4939447

[B70] Michel C. M. Murray M. M. Lantz G. Gonzalez S. Spinelli L. de Peralta R. G. (2004). EEG source imaging. Clin. Neurophysiol. 115, 2195–2222. 10.1016/j.clinph.2004.06.001 15351361

[B71] Moran R. Kiebel S. Stephan K. Reilly R. Daunizeau J. Friston K. (2007). A neural mass model of spectral responses in electrophysiology. Neuroimage 37, 706–720. 10.1016/j.neuroimage.2007.05.032 17632015 PMC2644418

[B72] Muldoon S. F. Pasqualetti F. Gu S. Cieslak M. Grafton S. T. Vettel J. M.. (2016). Stimulation-based control of dynamic brain networks. PLoS Comput. Biol. 12, e1005076. 10.1371/journal.pcbi.1005076 27611328 PMC5017638

[B73] Onslow A. C. Jones M. W. Bogacz R. (2014). A canonical circuit for generating phase-amplitude coupling. PLoS ONE 9, e102591. 10.1371/journal.pone.0102591 25136855 PMC4138025

[B74] Parkes L. Moore T. M. Calkins M. E. Cieslak M. Roalf D. R. Wolf D. H.. (2021). Network controllability in transmodal cortex predicts positive psychosis spectrum symptoms. Biol. Psychiatry 90, 409–418. 10.1016/j.biopsych.2021.03.016 34099190 PMC8842484

[B75] Pereira I. Frassle S. Heinzle J. Schobi D. Do C. T. Gruber M.. (2021). Conductance-based dynamic causal modeling: a mathematical review of its application to cross-power spectral densities. Neuroimage 245, 118662. 10.1016/j.neuroimage.2021.118662 34687862

[B76] Pinotsis D. A. Moran R. J. Friston K. J. (2012). Dynamic causal modeling with neural fields. Neuroimage 59, 1261–1274. 10.1016/j.neuroimage.2011.08.020 21924363 PMC3236998

[B77] Preti M. G. Van De Ville D. (2019). Decoupling of brain function from structure reveals regional behavioral specialization in humans. Nat. Commun. 10, 4747. 10.1038/s41467-019-12765-7 31628329 PMC6800438

[B78] Rabuffo G. Fousek J. Bernard C. Jirsa V. (2021). Neuronal cascades shape whole-brain functional dynamics at rest. eNeuro 8. 10.1523/ENEURO.0283-21.2021 34583933 PMC8555887

[B79] Raj A. Cai C. Xie X. Palacios E. Owen J. Mukherjee P.. (2020). Spectral graph theory of brain oscillations. Hum. Brain Mapp. 41, 2980–2998. 10.1002/hbm.24991 32202027 PMC7336150

[B80] Ranasinghe K. Verma P. Cai C. Xie X. Kudo K. Gao X.. (2022). Altered excitatory and inhibitory neuronal subpopulation parameters are distinctly associated with tau and amyloid in Alzheimer's disease. eLife 11, e77850. 10.7554/eLife.77850 35616532 PMC9217132

[B81] Razi A. Friston K. J. (2016). The connected brain: Causality, models, and intrinsic dynamics. IEEE Signal Process. Mag. 33, 14–35. 10.1109/MSP.2015.248212127038232

[B82] Ritter P. Schirner M. McIntosh A. R. Jirsa V. K. (2013). The virtual brain integrates computational modeling and multimodal neuroimaging. Brain Connect. 3, 121–145. 10.1089/brain.2012.0120 23442172 PMC3696923

[B83] Robinson P. A. Zhao X. Aquino K. M. Griffiths J. D. Sarkar S. Mehta-Pandejee G. (2016). Eigenmodes of brain activity: neural field theory predictions and comparison with experiment. Neuroimage 142, 79–98. 10.1016/j.neuroimage.2016.04.050 27157788

[B84] Sanz Perl Y. Pallavicini C. Perez Ipina I. Demertzi A. Bonhomme V. Martial C.. (2021). Perturbations in dynamical models of whole-brain activity dissociate between the level and stability of consciousness. PLOS Comput. Biol. 17, e1009139. 10.1371/journal.pcbi.1009139 34314430 PMC8315553

[B85] Sanz-Leon P. Knock S. A. Spiegler A. Jirsa V. K. (2015). Mathematical framework for large-scale brain network modeling in the virtual brain. Neuroimage 111, 385–430. 10.1016/j.neuroimage.2015.01.002 25592995

[B86] Scheid B. H. Ashourvan A. Stiso J. Davis K. A. Mikhail F. Pasqualetti F.. (2021). Time-evolving controllability of effective connectivity networks during seizure progression. Proc. Natl. Acad. Sci. U.S.A. 118, e2006436118. 10.1073/pnas.2006436118 33495341 PMC7865160

[B87] Shine J. M. Aburn M. J. Breakspear M. Poldrack R. A. (2018). The modulation of neural gain facilitates a transition between functional segregation and integration in the brain. eLife 7, e31130. 10.7554/eLife.31130 29376825 PMC5818252

[B88] Shine J. M. Müller E. J. Munn B. Cabral J. Moran R. J. Breakspear M. (2021). Computational models link cellular mechanisms of neuromodulation to large-scale neural dynamics. Nat. Neurosci. 24, 765–776. 10.1038/s41593-021-00824-6 33958801

[B89] Singh M. F. Braver T. S. Cole M. W. Ching S. (2020). Estimation and validation of individualized dynamic brain models with resting state fMRI. Neuroimage 221, 117046. 10.1016/j.neuroimage.2020.117046 32603858 PMC7875185

[B90] Sotero R. C. (2016). Topology, cross-frequency, and same-frequency band interactions shape the generation of phase-amplitude coupling in a neural mass model of a cortical column. PLoS Comput. Biol. 12, e1005180. 10.1371/journal.pcbi.1005180 27802274 PMC5089773

[B91] Srivastava P. Fotiadis P. Parkes L. Bassett D. S. (2022). The expanding horizons of network neuroscience: from description to prediction and control. Neuroimage 258, 119250. 10.1016/j.neuroimage.2022.119250 35659996 PMC11164099

[B92] Stiso J. Khambhati A. N. Menara T. Kahn A. E. Stein J. M. Das S. R.. (2019). White matter network architecture guides direct electrical stimulation through optimal state transitions. Cell Rep. 28, 2554.e7–2566.e7. 10.1016/j.celrep.2019.08.008 31484068 PMC6849479

[B93] Suarez L. E. Markello R. D. Betzel R. F. Misic B. (2020). Linking structure and function in macroscale brain networks. Trends Cogn. Sci. 24, 302–315. 10.1016/j.tics.2020.01.008 32160567

[B94] Tang E. Bassett D. S. (2018). Colloquium: control of dynamics in brain networks. Rev. Mod. Phys. 90, 031003. 10.1103/RevModPhys.90.031003

[B95] Tewarie P. Abeysuriya R. Byrne A. O'Neill G. C. Sotiropoulos S. N. Brookes M. J.. (2019). How do spatially distinct frequency specific MEG networks emerge from one underlying structural connectome? The role of the structural eigenmodes. Neuroimage 186, 211–220. 10.1016/j.neuroimage.2018.10.079 30399418

[B96] Tewarie P. Prasse B. Meier J. Byrne Á. De Domenico M. Stam C. J.. (2021). Interlayer connectivity reconstruction for multilayer brain networks using phase oscillator models. N. J. Phys. 23, 063065. 10.1088/1367-2630/ac066d

[B97] Tewarie P. Prasse B. Meier J. Santos F. Douw L. Schoonheim M.. (2020). Mapping functional brain networks from the structural connectome: relating the series expansion and eigenmode approaches. Neuroimage 216, 116805. 10.1016/j.neuroimage.2020.116805 32335264

[B98] Tewarie P. Prasse B. Meier J. Mandke K. Warrington S. Stam C. J.. (2022). Predicting time-resolved electrophysiological brain networks from structural eigenmodes. Hum. Brain Mapp. 10.1002/hbm.25967 35642600 PMC9435022

[B99] Tivadar R. I. Murray M. M. (2019). A primer on electroencephalography and event-related potentials for organizational neuroscience. Organ. Res. Methods 22, 69–94. 10.1177/1094428118804657

[B100] Verma P. Nagarajan S. Raj A. (2022a). Spectral graph theory of brain oscillations-revisited and improved. Neuroimage 249, 118919. 10.1016/j.neuroimage.2022.118919 35051584 PMC9506601

[B101] Verma P. Nagarajan S. Raj A. (2022b). Stability and dynamics of a spectral graph model of brain oscillations. Netw. Neurosci. 1–43. 10.1162/netn_a_00263 37334000 PMC10270709

[B102] Wang P. Kong R. Kong X. Liegeois R. Orban C. Deco G.. (2019). Inversion of a large-scale circuit model reveals a cortical hierarchy in the dynamic resting human brain. Sci. Adv. 5, eaat7854. 10.1126/sciadv.aat7854 30662942 PMC6326747

[B103] Wilmskoetter J. He X. Caciagli L. Jensen J. H. Marebwa B. Davis K. A.. (2022). Language recovery after brain injury: a structural network control theory study. J. Neurosci. 42, 657–669. 10.1523/JNEUROSCI.1096-21.2021 34872927 PMC8805614

[B104] Wilson H. R. Cowan J. D. (1972). Excitatory and inhibitory interactions in localized populations of model neurons. Biophys. J. 12, 1–24. 10.1016/S0006-3495(72)86068-5 4332108 PMC1484078

[B105] Wilson H. R. Cowan J. D. (1973). A mathematical theory of the functional dynamics of cortical and thalamic nervous tissue. Kybernetik 13, 55–80. 10.1007/BF00288786 4767470

[B106] Xie X. Cai C. Damasceno P. F. Nagarajan S. S. Raj A. (2021). Emergence of canonical functional networks from the structural connectome. Neuroimage 237, 118190. 10.1016/j.neuroimage.2021.118190 34022382 PMC8451304

[B107] Xie X. Kuceyeski A. Shah S. A. Schiff N. D. Nagarajan S. Raj A. (2019). Parameter identifiability and non-uniqueness in connectome based neural mass models. bioRxiv 480012. 10.1101/480012

[B108] Xie X. H. Stanley M. Damasceno P. F. (2020). Raj-Lab-UCSF/Spectrome: Spectral Graph Model of Neural Dynamics on Connectomes.

[B109] Yang G. J. Murray J. D. Wang X. -J. Glahn D. C. Pearlson G. D. Repovs G.. (2016). Functional hierarchy underlies preferential connectivity disturbances in schizophrenia. Proc. Natl. Acad. Sci. U.S.A. 113, E219–E228. 10.1073/pnas.1508436113 26699491 PMC4720350

[B110] Yeung M. S. Strogatz S. H. (1999). Time delay in the kuramoto model of coupled oscillators. Phys. Rev. Lett. 82, 648. 10.1103/PhysRevLett.82.648 21405399

[B111] Zamani Esfahlani F. Faskowitz J. Slack J. Mišić B. Betzel R. F. (2022). Local structure-function relationships in human brain networks across the lifespan. Nat. Commun. 13, 1–16. 10.1038/s41467-022-29770-y 35440659 PMC9018911

[B112] Zimmermann J. Perry A. Breakspear M. Schirner M. Sachdev P. Wen W.. (2018). Differentiation of Alzheimer's disease based on local and global parameters in personalized Virtual Brain models. Neuroimage Clin. 19, 240–251. 10.1016/j.nicl.2018.04.017 30035018 PMC6051478

[B113] Zoller D. Sandini C. Schaer M. Eliez S. Bassett D. S. Van De Ville D. (2021). Structural control energy of resting-state functional brain states reveals less cost-effective brain dynamics in psychosis vulnerability. Hum. Brain Mapp. 42, 2181–2200. 10.1002/hbm.25358 33566395 PMC8046160

