# Supplementary material for: Structure-function models of temporal, spatial, and spectral characteristics of non-invasive whole brain functional imaging
Source: Front Neurosci. 2022 Aug 30;16:959557. doi: 10.3389/fnins.2022.959557 (PMC9468900; doi:10.3389/fnins.2022.959557)
Supplement: Supplementary file 1 [file Data_Sheet_1.PDF]

Supplementary Material

We used a dual annealing optimization procedure in Python for parameter optimization Xiang et al. (1997). The dual annealing optimization was performed for three different initial guesses, and the parameter set leading to maximum sum of spectral and spatial correlations was chosen for each subject. The dual annealing settings were: `maxiter = 500`. All the other settings were the same as default. Parameter initial guesses and bounds for estimating the static spectra are specified in Table S1.

Table S1. SGM parameter values, initial guesses, and bounds for parameter estimation for static spectra fitting

**Name**	**Symbol**	**Initial value 1**	**Initial value 2**	**Initial value 3**	**Lower/upper bound for optimization**
Excitatory time constant	τ_e	0.015 s	0.025 s	0.006 s	[0.005 s, 0.03 s]
Inhibitory time constant	τ_i	0.01 s	0.08 s	0.15 s	[0.005 s, 0.2 s]
Long-range connectivity coupling constant	α	1	0.5	0.1	[0.1, 1]
Transmission speed	v	1 m/s	5 m/s	8 m/s	[0.001 m/s, 10 m/s]
Alternating population gain	g_{ei}	8	5	1	[0.5, 10]
Inhibitory gain	g_{ii}	1	5	8	[0.5, 10]
Graph time constant	τ_G	0.006 s	0.015 s	0.025 s	[0.005 s, 0.03 s]
Excitatory gain	g_{ee}	n/a	n/a	n/a	n/a

REFERENCES

Xiang, Y., Sun, D., Fan, W., and Gong, X. (1997). Generalized simulated annealing algorithm and its application to the Thomson model. *Physics Letters A* 233, 216–220. doi:https://doi.org/10.1016/S0375-9601(97)00474-X
